# Use of Dexmedetomidine as an Adjuvant in Spinal Anesthesia in Patients With Femoral Fracture Affected by Moderate Aortic Stenosis: A Case Series

**DOI:** 10.7759/cureus.83206

**Published:** 2025-04-29

**Authors:** Stefano Barbaro, Ester Scapini, Pierdomenico Carone, Laura Lanotte, Antonio Zagaria, Gennaro Gadaleta Caldarola, Michele Debitonto

**Affiliations:** 1 Anesthesia, Resuscitation, and Pain Therapy, Ospedale Monsignor Dimiccoli, Barletta, ITA; 2 Oncology, Ospedale Monsignor Dimiccoli, Barletta, ITA

**Keywords:** adjuvant drugs, `anesthesia, anesthesia spinal, critical aortic stenosis, elderly population

## Abstract

The anaesthesiological management of femoral fracture in the frail patient still represents a challenge for the anaesthetist and his team. For decades, the most frequently used anaesthesiological approach in these cases has been spinal anaesthesia. In recent years, the progression of pharmacology and anaesthesiological techniques has meant that this technique can be adopted in situations where general anaesthesia was previously preferred, particularly in clinical scenarios requiring less haemodynamic impact, such as in patients with aortic valve stenosis. In this case series, we consider how adjuvant spinal anaesthesia with dexmedetomidine ensures adequate intra- and post-operative analgesia and haemodynamic stability in patients with moderate aortic valvular stenosis. Specifically, we used the Visual Analogue Scale (VAS) score to evaluate pain, both intraoperatively and postoperatively at 6, 12, and 24 hours.

## Introduction

Aortic stenosis is a narrowing of the aortic valve that obstructs the flow of blood from the left ventricle to the aorta. Aortic stenosis has an initial latent period, during which progressive worsening of left ventricular (LV) outflow obstruction leads to compensatory hypertrophic changes in the LV myocardium [1]. The resultant increase in LV systolic function helps maintain adequate systemic pressures [2]. Previously, general anesthesia (GA) has been used as the anesthetic technique of choice for femoral fracture surgery. The modern literature shows good results, but the evidence is still not robust regarding hemodynamic stability with neuraxial anesthesia management in patients with moderate aortic stenosis [3], especially due to the use of combined anesthesia techniques. Furthermore, the use of adjuvant drugs with local anesthetics in neuraxial anesthesia helps to prolong the anesthetic effect, and therefore, in our case, contributes to the goal of reducing the use of painkillers in these patients with cardiovascular problems.

## Case presentation

We selected three patients for surgery for medial femoral fracture. In agreement with the patients and their relatives, and after explaining the anesthesiological risks, it was decided to use an anesthesiological technique that did not include GA but could prolong postoperative analgesia while minimizing the use of anti-inflammatory drugs, given the patients' various conditions. As these were elderly patients with cardiac and pulmonary problems, we preferred to avoid the use of sedative and hypnotic drugs in the intra- and postoperative periods.

Patient 1 is an 83-year-old with a left femur fracture, classified as American Society of Anesthesiologists (ASA) physical status 3, with a history of chronic obstructive pulmonary disease (COPD), arterial hypertension, Parkinson’s disease, BMI 35 kg/m², ex-smoker, and hypercholesterolemia, with moderate-to-severe aortic stenosis already treated with transcatheter aortic valve implantation (TAVI). Preoperatively, a cardiological evaluation highlighted the presence of aortic stenosis with a valve area of 1.25 cm² and a mean gradient of 41 millimeters of mercury (mmHg). In the operating room, heart rate (HR), invasive blood pressure, continuous electrocardiogram, and oxygen saturation were recorded before starting. One thousand milliliters (mL) of Ringer lactate was administered in the preoperative period. An ultrasound-guided femoral nerve block was performed with 15 mL of levobupivacaine 0.375%, followed by non-selected spinal anesthesia with levobupivacaine 0.25% (4 mL) and dexmedetomidine 10 micrograms (mcg), for a total volume of 5 mL. The same anesthetic protocol was used in all three patients. We noted motor block for about 3 hours; VAS scores were 0 at 6 hours, 2 at 12 hours, and 0 at 24 hours. Paracetamol was requested by the patient after about 5 hours. No postoperative complications were noted.

Patient 2, 81 years old, with a left femur fracture, ASA 3, had a history of COPD, Alzheimer’s disease, obesity (BMI 32 kg/m²), type 2 diabetes mellitus, and was a smoker, with moderate aortic stenosis. Preoperatively, a cardiological evaluation revealed a valve area of 1.4 cm² and a mean gradient of 40 mmHg. Motor block lasted about 3 hours; VAS scores were 3 at 6 hours, 2 at 12 hours, and 0 at 24 hours. Paracetamol was requested after about 6 hours. No postoperative complications were noted.

Patient 3 is an 83-year-old with a right femur fracture, ASA 3, with a history of COPD, obesity (BMI 29 kg/m²), ex-smoker, and type 2 diabetes mellitus, with moderate aortic stenosis already treated with TAVI. Preoperatively, a cardiological evaluation revealed a valve area of 1.5 cm² and a mean gradient of 37 mmHg. Motor block lasted about 3 hours; VAS scores were 2 at 6 hours, 0 at 12 hours, and 0 at 24 hours. Paracetamol was requested after about 5 hours. No postoperative complications were noted.

## Discussion

Aortic valvular stenosis is the most common acquired heart valve disease in the Western world, with a prevalence of >2% in individuals over 60 years of age, but more importantly, it carries a mortality rate of 50% at two years in patients symptomatic for this disease [4]. The goal of the anesthesia strategy is to have the least hemodynamic impact and, like all anesthetic techniques, to guarantee the safety of the patient.

The main issue is that continuous cardiac mechanical stress can, over time, lead to the onset of cardiac remodeling, with subsequent hemodynamic instability, worsening of symptoms, and deterioration of the general clinical picture [5].

From a hemodynamic point of view, in these patients, aortic stenosis increases the afterload, resulting in hypertrophy of the left ventricular myocytes and therefore anatomical remodeling [6]. This situation leads to a reduction in myocardial perfusion secondary to the supply/demand discrepancy, with subsequent cell death, activation of the renin-angiotensin-aldosterone axis, and activation of fibroblasts within the cardiac wall, resulting in myocardial interstitial fibrosis [6].

The gold standard for diagnosis is transthoracic echocardiography, with moderate valvular stenosis defined by an aortic valve area between 1 and 1.5 cm², or body surface area-indexed valve area between 0.6 and 0.9 cm²/m², a transvalvular pressure gradient (TPG) between 25 and 40 mmHg, or a maximum aortic jet velocity between 3 and 4 m/sec [7].

It is important to note that although spinal anesthesia is generally considered safe, there are still some risks and potential side effects.

Spinal anesthesia mainly affects hemodynamics. Carpenter RL et al. [8] showed that patients undergoing spinal anesthesia developed arterial hypotension (systolic blood pressure <90 mmHg) in 33% of cases, bradycardia (<50 bpm) in 13%, and cardiac arrhythmias in 2%.

In elderly patients, hypotension after spinal anesthesia appears to be more pronounced due to two factors: 1) a greater impact on a more reactive resting sympathetic nervous system; and 2) reduced baroreceptor activity. As a result, there is typically a reduction in systemic vascular resistance (SVR), cardiac output (CO), or both [9].

Dexmedetomidine is a selective alpha-2 adrenergic receptor agonist with sedative and analgesic effects [10], and recent scientific studies appear to show optimization, decreased onset time, and prolonged analgesic effects when used as an adjuvant to local anesthetics in the subarachnoid and perineural spaces [11,12].

This synergy between dexmedetomidine and local anesthetics could represent an important step forward in perioperative pain management, improving not only patient comfort during surgery but also the quality of postoperative recovery. The use of dexmedetomidine could reduce the need for opioid analgesics, helping to minimize the side effects associated with these drugs, such as respiratory depression and excessive sedation. Of course, if there is no consent from the patient, or if there are contraindications to the execution of spinal anesthesia, another anesthesiological method must necessarily be adopted. Furthermore, dexmedetomidine’s ability to provide deep and controlled sedation without compromising the patient's hemodynamic stability makes it a particularly attractive option in complex surgical settings.

In this opioid-free anesthesia approach, it is easy to integrate into the Enhanced Recovery After Surgery (ERAS) protocol program, optimizing hospitalization outcomes [13].

## Conclusions

The use of spinal anesthesia in patients with moderate aortic stenosis is not an absolute contraindication. Today, we have anesthetic techniques available that, when combined, can reduce the hemodynamic impact as well as prolong the postoperative analgesic effect in selected patients in whom minimizing the use of anti-inflammatory drugs is desirable. It would be useful to develop randomized controlled trials (RCTs) to evaluate this approach, including comparisons of the use of dexmedetomidine as an adjuvant in spinal anesthesia alongside local anesthetics, and also in combination with different concentrations of local anesthetic. Given the small number of patients with aortic stenosis who are candidates for interventions such as the one described in this study, a multicenter collaboration to develop a possible RCT would be desirable.
